# Comparison Between Three-Dimensional Diffusion-Weighted PSIF Technique and Routine Imaging Sequences in Evaluation of Peripheral Nerves in Healthy People

**DOI:** 10.29252/NIRP.BCN.9.1.65

**Published:** 2018

**Authors:** Mahsa Zare, Fariborz Faeghi, Ashrafsadat Hosseini, Mohammad Sobhan Ardekani, Mohammad Hossein Heidari, Ehsan Zarei

**Affiliations:** 1. Department of Radiology Technology, School of Allied Medical Sciences, Shahid Beheshti University of Medical Sciences, Tehran, Iran.; 2. Department of Radiology, Shahid Sadoughi Hospital, Shahid Sadoughi University of Medical Sciences, Yazd, Iran.; 3. Department of Basic Sciences, School of Allied Medical Sciences, Shahid Beheshti University of Medical Sciences, Tehran, Iran.; 4. Department of Physical Education, School of Education & Psychology, Shiraz University, Shiraz, Iran.

**Keywords:** MR neurography, 3D DW PSIF, Peripheral nerves, Lumbosacral plexus, Brachial plexus

## Abstract

**Introduction:**

The present study aims to evaluate the Three-Dimensional Diffusion-Weighted reversed fast imaging with steady state free precession (3D DW-PSIF) sequence with respect to imaging of the peripheral nerves; the tibial, medial, and lateral plantar nerves in the lower extremity, ulnar and median nerve in the upper extremity, sciatic nerve, brachial plexus, and lumbosacral plexus, and also to compare its usefulness with the current two-dimensional sequences on a 1.5 T MR scanner.

**Methods:**

A total of 25 healthy subjects underwent MR imaging of peripheral nerves, 5 subjects in each area. In each imaging sequence, including T2W SPAIR and 3D DW-PSIF, images were evaluated for ability to identify the nerves in the related area using a 3-score scale (0–2). Then, by summing up the conspicuity scores, a total certainty score was recorded for each sequence.

**Results:**

With combining the results of all studies, the conspicuity mean (SD) score was 1.57(0.67) on the 3D DW-PSIF images, and 0.74(0.76) on the T2-weighted images (P<0.001). Regarding the lumbosacral plexus, the corresponding certainty mean (SD) scores were 1.80(0.40) and 1.07(0.74) (P<0.001) and with regard to the brachial plexus, they were 1.23(0.83) and 0.75(0.84), (P<0.001). Regarding the ankle/hind foot they were 1.87(0.35) and 0.40(0.50) (P<0.001) and in the wrist/proximal hand, 1.70(0.48) and 0.50(0.52) (P<0.001). Regarding the sciatic nerve, they were 1.80(0.44) and 0.20(0.44) (P=0.003).

**Conclusion:**

3D DW PSIF provides better manifestation of nerves compared to routine imaging sequences particularly fat saturated T2W images. This novel imaging technique can be used in MR neurography examination protocol for exact localization of the nerve and evaluation of the nerve pathology.

## 1. Introduction

There are various methods for the evaluation of peripheral nerves. Clinical examinations and electrodiagnostic tests are traditional methods for evaluating the peripheral nerves. In most cases, these methods provide incomplete information about the anatomic details and the nerve intensity (Chhabra et al., 2011a; Chhabra et al., 2013). Sonography is a complementary method used to evaluate the peripheral nerves injury. However, this method has also certain restrictions, for example its dependency on a well-experienced and knowledgeable operator with the pretentious sonography of the soft tissues structures (Khachi, Skirgaudes, Lee, & Wollstein, 2007).

MR imaging of the nerves, called Magnetic Resonance Neurography, is an advanced technique for the evaluation of normal and abnormal peripheral nerves (Chhabra et al., 2011c). It mostly benefits from the techniques of two-dimensional MR imaging (Zhang et al., 2008a). Two-dimensional images have advantages such as high SNR, fairly short acquisition time, and radiolo-gist’s familiarity with it, however, it barely distinguishes between small peripheral nerves and adjacent vascular structures due to their similar size and signal intensity and also performs poorly during saturation of subcutaneous and fascial edema, particularly in elbow and knee (Chhabra, et al., 2011c; Chhabra, Subhawong, Bizzell, Flammang, & Soldatos, 2011d).

Three-dimensional diffusion-weighted PSIF (3D-DWPSIF) sequence can help cope with such restrictions and obtain images which represent detailed anatomy and accurate localization of the nerves. The steady state nature of this sequence, and use of diffusion gradient, leads to saturation of water signal and vascular structure. Therefore, small nerves can be distinguished from the adjacent vessels more effectively (Chhabra, et al., 2011c; Chhabra et al., 2011d; Chhabra, Lee, Bizzell, & Soldatos, 2011b). The current study aims to evaluate the 3D DW-PSIF sequence in imaging peripheral nervous system including the nerves in the distal lower and upper extremities, sciatic nerve, brachial plexus, and lumbosacral plexus, and also to compare its results with the current two-dimensional sequences which utilize 1.5 Tesla magnet.

## 2. Methods

A total of 25 healthy subjects (15 males and 10 females, mean (SD) age; 32.5(35) years; age range; 20–55 years) participated voluntarily in the study. We took written consent forms from all study participants. MR imaging of each part of peripheral nerves was done in 5 subjects. Totally, 100 peripheral nerves were analyzed as follows: C1 to C8 nerves of brachial plexus; L1 to S1 nerves of lumbosacral plexus; Median and ulnar nerves in wrist and palm; Tibial, medial, and lateral plantar nerves in the ankle and hind foot; and Sciatic nerve and it bifurcation.

Images were taken by a 1.5 T MR scanner (Avanto, Siemens Medical Solutions, Erlangen, Germany) using body matrix coil, neck matrix coil, head matrix coil, and CP flexible coil. This study used T2-weighted Spectrally Adiabatic Inversion Recovery (SPAIR) sequence (TR/TE=2500-6700/36-100 ms, Slice Thickness=2.5–4), as well as high-resolution 3D-DWI-PSIF sequence with dominant T2 contrast. The 3D DW-PSIF sequence was primarily optimized for peripheral nerve imaging in each part of the body with the change in sequence parameters according to Table 1.

**Table 1.** The acquired parameters of the 3D DW-PSIF sequence

**Acquired Parameters**	**Value**
Slabs	1
Matrix size	256–256
Field of view	170–300 ; 100 mm
Slice thickness	0.7–1 mm
TR	10 ms
TE	2.47 ms
Averages	2
Flip angle	35 Degrees
Fat suppression	Water excitation normal
Diffusion mode	Phase, read
Diffusion directions	1
Dimension	3D
3D partitions	50–90
Effective spatial resolution	0.7–1.2 ; 0.7–1.2 ; 0.7–1 mm
B-value	90–100 s/mm^2^
Elliptical scanning	On
Asymmetric echo	Off
Receiver bandwidth	40 Hz/Px
Acquisitions	1
Time of acquisition	5 min 49 s-7 min 28 s

In the 3D DW-PSIF sequence, water excitation method was used for fat suppression. In this sequence, low diffusion moment (80–90 s/mm^2^) was applied in the appropriate direction (phase encoding, frequency encoding, or slice selection) depending on the related area for imaging. Furthermore, flow compensated gradient was applied in readout direction, in order to reduce the flow artifact. In lumbosacral and brachial plexus, the phase direction was in feet to head. In extremities and sciatic nerve, the phase direction was in anteroposterior direction. The diffusion moment was applied along the phase encoding direction in extremities, sciatic nerve, and brachial plexus, but in lumbosacral plexus, applied along the readout direction.

Imaging was done on coronal or sagittal planes to keep the acquisition time low in all studies. Therefore, with using these parameters, we selected images of the nerves with characteristics such as uniform fat, resulting in good nerve contrast to the background, and effective suppression of the vessels (Chhabra et al., 2011c). Then, 3D DW-PSIF images were reformatted with appropriate thickness using postprocessing techniques such as MPR and MIP. Since this sequence was isotropic, the nerve could be reformatted in any arbitrary plane (Chhabra et al., 2011c). The thickness of the reconstructed slab, varies between 3 to 20 mm depending on the nerve size in the related area.

### 2.1. Image analysis and statistical approaches

Two experienced radiologists evaluated the images on a Picture Archiving and Communication System (PACS) workstation. In each study, the T2W and 3D DW-PSIF images were analyzed separately. In each imaging sequence, images were evaluated for the ability to represent the nerves in the related area, uniformly fat saturation, and suppress signal from the vessels. Each radiologist independently performed primary evaluation of the images, but final conclusion about the scores given to each sequence was done with consultation.

The conspicuity of each nerve was determined using a semi-quantitative scale (0–2) as follows: 2: Nerve identified with certainty; 1: Nerve probably identified; 0: Nerve not identified. Then, by summing up the conspicuity scores of all evaluated nerves in the related images, a total conspicuity score was recorded for each imaging sequence. In order to evaluate differences in conspicuity scores between sequences in each study, paired samples t test was performed. A probability level of 0.05 was considered as statistically significant.

## 3. Results

Table 2 presents the study results. In the current study, 3D DW-PSIF sequence was investigated in most peripheral nerves including lumbosacral plexus, brachial plexus, median and ulnar nerves in the upper limb, tibial, medial and lateral plantar nerves in the lower limb, and sciatic nerve. Furthermore, routine sequences such as T1W-axial images, and T2W-axial and coronal images with fat saturation via SPAIR method were also used. In the 3D DW-PSIF sequence, small nerves and adjacent vessels were better distinguished compared with T2W sequence, and fat saturation was done more effectively than T2W SPAIR method.

**Table 2.** Summary of the conspicuity scores obtained from the imaging sequences

	**3D-DW-PSIF Images**	**T2W Images**	**P**
MR neurography studies of the lumbosacral plexus (n=5)			
Nerves identified with certainty	0	7	-
Nerves probably identified	6	14	-
Nerves not identified	24	9	-
Conspicuity mean±SD score	1.80±0.40	1.07±0.74	<0.001
MR neurography studies of the brachial plexus (n=5)			
Nerves identified with certainty	10	20	-
Nerves probably identified	11	10	-
Nerves not identified	19	10	-
Conspicuity mean±SD score	1.23±0.83	0.75±0.84	<0.001
MR neurography studies of the ankle/hind foot (n=5)			
Nerves identified with certainty	0	9	-
Nerves probably identified	2	6	-
Nerves not identified	13	0	-
Conspicuity mean±SD score	1.87±0.35	0.40±0.50	<0.001
MR neurography studies of the wrist/proximal hand (n=5)			
Nerves identified with certainty	0	5	-
Nerves probably identified	3	5	-
Nerves not identified	7	2	-
Conspicuity mean±SD score	1.70±0.48	0.50±0.52	<0.001
MR neurography studies of the sciatic nerve (n=5)			
Nerves identified with certainty	0	4	-
Nerves probably identified	1	1	-
Nerves not identified	4	0	-
Conspicuity mean±SD score	1.80±0.44	0.20±0.44	0.003
All MR neurography studies (n=25)			
Nerves identified with certainty	10	45	-
Nerves probably identified	23	36	-
Nerves not identified	67	19	-
Conspicuity mean±SD score	1.57±0.67	0.74±0.76	<0.001

MR: Magnetic Resonance; 3D DW-PSIF: 3-Dimensional Diffusion-Weighted MR based on reversed fast imaging with steady state precession; T2W: T2-weighted

All conspicuity score values are expressed as average value±standard deviation.

**Figure 1.**
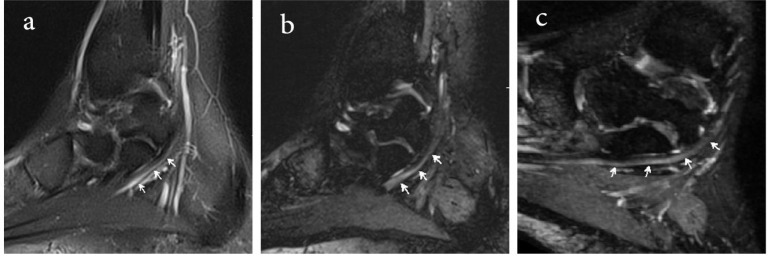
MR neurography of the distal lower extremity in a 26-year-old male The identification of the medial plantar nerve (arrows) is poor on the sagittal fat-suppressed T2-weighted image due to adjacent T2 hyperintense vessels (a), and the conspicuity score is 1. In the 3D DW-PSIF image (b), the identification of the medial plantar nerves (arrows) is definite, and the conspicuity scores is 2. In the isotropic reconstructed oblique sagittal (c) image, the medial planter nerve (arrows) can be easily depicted.

**Figure 2.**
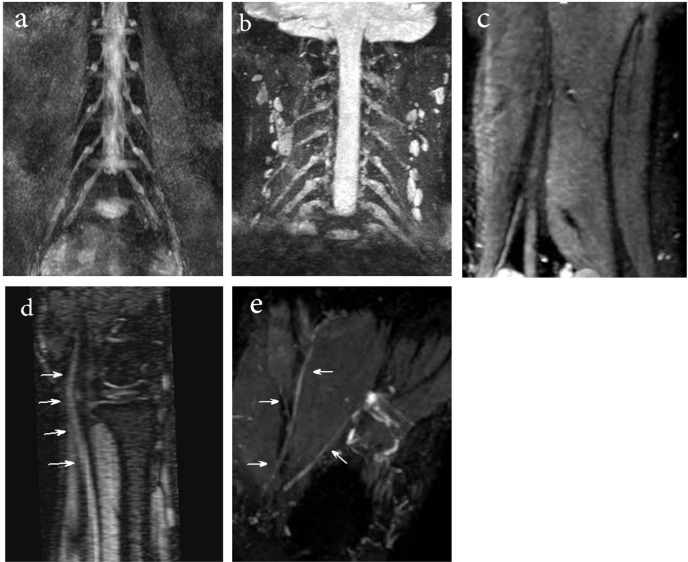
Thin section MIP reconstruction MR images, obtained with a 3D DW-SSFP sequence a: Lumbosacral plexus; b: Normal nerve root ganglia and proximal part of brachial plexus; c: Sciatic nerve and it branches; d: Median nerve; e: Medial and lateral plantar nerve

Of 100 analyzed peripheral nerves, 67% of the nerves on 3D-DW-PSIF images and 19% of the nerves on T2W images were recognized with certainty. About 45% of the peripheral nerves on T2W images were not identified. In all study MR examinations, the conspicuity scores corresponding to 3D DW-PSIF images were higher than those corresponding to T2W images (Figure 1). In conclusion, the 3D-DW-PSIF sequence performs very well in representing the nerves including ulnar and median nerve, tibial, medial and lateral plantar nerve, and sciatic nerve. Furthermore, this sequence represents the nerves in lumbosacral plexus quite clearly (Figure 2). This can be due to excellent fat suppression in these areas and saturation of signals from the vessels and fluids because the steady state nature of this sequence and also employing diffusion gradients in the appropriate direction.

In the brachial plexus imaging, due to large FOV and accordingly suboptimal or no fat saturation in off-center areas of the neck, 3D DW-PSIF is only able to represent nerve roots ganglia and proximal part of brachial plexus (Figure 1). Therefore to obtain high quality images of brachial plexus with uniform fat suppression, other three-dimensional sequences, such as 3D-STIR-SPACE can be used.

## 4. Discussion

With recent advances in MR imaging of peripheral nerves, high quality images of the nerves can be obtained. So nerve anatomy and pathology is effectively depicted in these images (Bendszus et al., 2003; Howe, Filler, Bell, & Griffiths, 1992). These images can also be used in pre-operative assessments of nerve injury and entrapments (Chhabra et al., 2011c). The routine imaging techniques commonly used in the assessment of peripheral nerves include fat-saturated T2-weighted fast spin-echo, short inversion-recovery with fat saturation (STIR), and T2W spectral adiabatic inversion recovery turbo spin echo (T2 SPAIR TSE) sequences. Although these methods produce high-quality images, they have limitations in representing small peripheral nerves, because the peripheral nerves and vessels have similar diameter and T2 signal intensity resulting in poor discrimination (Zhang et al., 2008a; Chhabra et al., 2011c; Chhabra et al., 2011d). Furthermore, high-resolution T1-weighted Spin Echo Sequence can also be used to evaluate the fascicular structure of normal nerves characterized with abundant peri- and intra-mural fat that is dispersed between fascicles and also distinguish them from the adjacent vessels (Chhabra et al., 2010). However, since nerve injuries and entrapments lead to loss of this perineural fat in involved areas, T1W images are not usually helpful (Chhabra et al., 2011c; Chhabra et al., 2010).

3D-DW-SSFP sequence, used in this study, is a high resolution three dimensional diffusion weighted steady state free precession imaging in combination with fat saturation and flow compensation techniques (Zhang et al., 2008a). This new sequence has several advantages. The signal intensity is formed by spin echo mechanism with characteristics similar to a spin echo sequence, and therefore, has T2 dominant contrast. Hence, the effect of magnetic field inhomogeneities is reduced due to the spin relaxation (Hoffmann et al., 2000). Using continuous fine sections in this sequence, as well as application of high isotropic resolution, allows representation of small structures and injuries and makes it possible to reconstructive the images in any desired plane with good quality using maximum intensity projections technique (Zhang et al., 2008a).

Since peripheral nerves pass over various tortuous paths, an attempt to suppress vascular signal using saturation bands will lead to failure, particularly in extremities (Chhabra et al., 2011a; Chhabra et al., 2011c; Chhabra et al., 2010). The steady state nature of 3D-DW-PSIF sequence, effectively eliminates the signal of vascular flow (Taupitz et al., 1995; Zhang et al., 2008b). Furthermore, application of low diffusion moment (b 80–90 s/mm^2^) in this sequence is helpful in suppression of signals from the vessels (Chhabra et al., 2011a; Chhabra et al., 2011b; Chavhan, Babyn, Jankharia, Cheng, & Shroff, 2008). By using this parameters, we will be able to distinguish the high T2 signal intensity of the nerves from the nulled signal of adjacent vessels. Therefore in comparison with T2W sequence, 3D-DW-PSIF sequence provides better differentiation between small peripheral nerves and adjacent vessels (Chhabra et al., 2011c; Chhabra et al., 2011d). In addition, with using diffusion gradients, the subcutaneous and fascial edema that limits the evaluation of small peripheral nerves (in particular distal to elbow and knee), selectively suppresses and accordingly increases the relative nerve conspicuity (Chhabra et al., 2010; Baur & Reiser, 2000). Since water excitation technique is used in this sequence, the fat signal intensity is fully suppressed. Also chemical shift effects and motion dependent phase errors are reduced (Zhang et al., 2008a).

An important factor on 3D DW-PSIF imaging is its good shimming. The large FOV and off-center areas may lead to ghosting artifacts and non-uniform fat suppression such as in brachial plexus, which makes the assessment of these neural pathways problematic. The most significant restriction in this study is high sensitivity of 3D DWPSIF to motion artifact, breathing artifact and magnetic field inhomogeneity (Chhabra et al., 2011d; Chhabra, Flammang, Padua, Carrino, & Andreisek, 2014), which limits the image quality. Another restriction is low Signal to Noise Ratio (SNR) of this sequence in comparison with T2W sequence; as a result, fascicular structure of the nerves is always better represented in fat suppressed T2W. Therefore, 3D DW-PSIF may not replace the T2-weighted imaging which is commonly used in MR Neurography (Chhabra et al., 2011c; Chhabra et al., 2011d).

3D-DW-PSIF imaging was first successfully used for the evaluation of cranial nerves in healthy people by Zhang et al. in 2008. (Zhang et al., 2008b). The studies conducted in this respect have emphasized on the usefulness of this imaging method in the assessment of central and peripheral nerves in human body. In a study in 2008, the 3D DW-PSIF sequence used to determine structures of the human Lumbosacral Plexus (LSP). This study showed that this technique can clearly reveal detailed anatomy of the LSP and its branches (Zhang et al., 2008a). In 2010, this imaging method was used for visualization of the intraparotid facial nerve and parotid duct in 10 patients with suspected parotid gland disease. According to the results, the excellent fat suppression, high spatial resolution, vascular signal suppression, and sufficient T2-weighting of 3D-PSIF allowed for simultaneous visualization (Reimer, Parizel, Meaney, & Stichnoth, 2010). In another study in 2011, the researchers investigated the 3D-PSIF sequence in the evaluation of upper and lower extremities and compared its results with routine 2D sequence. They concluded that the 3D-PSIF images provide better identification of the nerves compared to the T2-weighted images (Chhabra et al., 2011c).

In this study, we used this new sequence in peripheral nerves imaging of distal lower and upper extremities, sciatic nerve, brachial plexus, and lumbosacral plexus. We found that the conspicuity scores in 3D DW-PSIF images were higher compared to corresponding scores in T2W images. In addition, 3D PSIF sequence provides better manifestation of nerves compared to fat-saturated T2W sequence. Our data suggest that this novel imaging sequence can be used in MR neurography examination protocol for exact localization of the nerve and evaluation of the nerve pathology.
